# Despite what we know, physical activity continues to decline: can integrating physical activity and education be a way forward?

**DOI:** 10.3389/fspor.2026.1832941

**Published:** 2026-08-31

**Authors:** Øyvind Sandbakk, Erik Grasaas

**Affiliations:** 1 School of Sport Science, UiT, the Artic University of Norway, Tromsø, Norway; 2 Department of Physical Performance, Norwegian School of Sport Sciences, Oslo, Norway; 3 Teacher Education Unit, University of Agder, Kristiansand, Norway

**Keywords:** adolescence, exercise habits, school, sports engagement, well-being

## Abstract

Despite decades of robust evidence on the benefits of regular physical activity, the fitness and health of young people are in worrying decline worldwide. Today, around 80% of adolescents fail to meet recommended daily activity levels, a trend which parallels an increase in youth overweight and obesity rates. This perspective paper examines the wide-ranging consequences of inactivity in adolescence, ranging from heightened long-term risks of chronic disease and rising healthcare burdens, to negative impacts on mental health, well-being, and the development of self-efficacy in adolescence. We discuss the possible promises and pitfalls of organized sport as a traditional strategy to engage youth in physical activity: while sports participation is linked to better physical and mental health outcomes, many adolescents seems to drop out of sport in their mid-teens due to factors such as early specialization, competitive pressures, cost, and social disparities. Therefore, we argue that youth inactivity will require a broader, more inclusive approach that goes beyond the sports club model, and propose integrating substantial daily physical activity into school curricula as a universal foundation for fitness and health. By reimagining schools as key platforms for physical activity—in partnership with community sports programs—and by implementing bold, multi-sector policies to reduce barriers to active lifestyles, society can begin to bridge the gap between what we know and what we practice. Translating knowledge into action is crucial to safeguard the health, well-being, and future resilience of the next generation.

## 1 Introduction

The physical fitness of modern youth is declining worldwide, with most adolescents now failing to meet basic activity guidelines. Global analyses indicate that roughly 80% of teenagers are not achieving the recommended 60 min of moderate-to-vigorous physical activity per day (1). This inactivity pattern coincides with increases in youth overweight: the World Health Organization (WHO) reports that the prevalence of overweight (including obesity) among 5–19-year-olds climbed from about 8% in 1990 to 20% in 2022 (2). In absolute terms, over 390 million young people globally were overweight in 2022, including 160 million classified as obese (2). These tendencies have occurred amidst increasingly sedentary lifestyles, characterized by greater screen time, academic pressures, and reduced opportunities for free play, which have collectively de-prioritized daily physical activity. These trends span high-income and low-income countries alike (3). Notably, girls are consistently less active than boys, a pattern observed across countries (1, 4). For example, a recent Norwegian survey of 13–19-year-olds found only 17%–20% of girls met activity recommendations, compared to roughly 28%–31% of boys (4). Social disparities in physical activity is an additional recurring theme: youth from lower socioeconomic backgrounds also tend to be less active and have higher drop-out rates from sports than their more affluent peers (5). However, declining participation in organized sport is likely multifactorial. In addition to organizational and socioeconomic influences, increasing screen time, digital entertainment, evolving leisure preferences, and broader cultural changes may also contribute to reduced participation in both organized sport and overall physical activity (6). In sum, despite strong evidence of the importance of physical activity for young people's health, such as increased protection against non-communicable diseases (7), the world is witnessing a broad decline in daily youth physical activity and fitness. This decline is closely intertwined with rising obesity rates and widening health inequalities on a global scale.

These trends are concerning and warrant attention: despite knowing how essential youth physical activity is for health and well-being, we have yet to reverse the downward trajectory in fitness. In this perspective paper, we explore the possible causes and consequences of that paradox and outlines potential solutions—examining the far-reaching impacts of widespread youth inactivity, evaluating the limitations of relying solely on organized sports, and proposing how our education systems can be harnessed to embed daily physical activity as a fundamental part of young people's lives.

## 2 Consequences of inactivity in youth

The consequences of declining youth fitness are far-reaching. Physically inactive lifestyles established in adolescence increase risks for numerous chronic diseases in adulthood, including cardiovascular disease, type 2 diabetes, and some cancers (3, 8). According to the WHO, if current trends continue, almost 500 million new cases of preventable non-communicable diseases could occur globally by 2030 due to physical inactivity, an outcome that would impose a staggering burden on healthcare systems (9). The economic costs of treating these largely avoidable conditions are estimated at around $300 billion in direct health expenditures by 2030, straining health budgets worldwide (9). In the long run, diminished youth fitness also affects workforces and national productivity through higher rates of sickness absence and lower labor force participation as this generation ages. Another impact is on military preparedness. In countries like the United States, military leaders warn that the poorer fitness of young people has become a national security concern. In 2018, about 71% of U.S. young people aged 17–24 would not qualify for military service, primarily due to issues related to obesity, poor fitness, or other health problems (10).

These statistics underscore how pervasive physical inactivity has societal consequences beyond personal health, affecting everything from healthcare costs to defense capabilities. Equally important are the quality-of-life implications: active youth generally report better mental health, higher life satisfaction, and stronger social connectedness than their inactive peers (11). Conversely, a less active youth population could mean more young people suffering from stress, low self-esteem, and social isolation. In Norway, for instance, adolescents who participated in sports demonstrated higher life satisfaction across almost all sports disciplines compared to national averages, with especially strong benefits seen among girls (12). If large segments of the next generation grow up unfit and disengaged from physical activity, the loss will be felt not only in epidemiological statistics and economic ledgers, but in the everyday well-being and resilience of the population.

Moreover, the lack of participation in physical activity inhibits opportunities to experience progress, overcome challenges, and develop competence in a tangible way. Self-efficacy, defined as the belief in one's capacity to organize and execute the actions required to achieve desired outcomes (13), is shown to be an underlying beneficial factor influencing physical activity levels in adolescence. In Norway, a population-based analysis among adolescents revealed that life satisfaction is explained not only by the level of physical activity but also by the mediating role of self-efficacy (14). This understanding is pivotal, because adolescents who regularly experience mastery through movement, sport, and physical challenges are more likely to develop a sense of capability that extends beyond the sporting context and into education, work, and broader life domains. Conversely, prolonged physical inactivity during adolescence limits opportunities for such experiences and weakens the development of self-belief. Hence, this has implications that reach beyond individual and public health: fostering physically active youth may contribute to a generation better equipped with confidence, resilience, and the belief that they can influence their own future and contribute constructively to society.

## 3 Organized sport as an instrument

Organized sport is often suggested as one of the solutions to reduce youth inactivity because sports participation can provide regular exercise, camaraderie, and motivation for young people to stay active. Indeed, numerous studies link sports involvement in adolescence to positive health and social outcomes, such as higher activity levels, lower stress, and improved mental health (11). In Norway, a cross-sectional analysis of many thousands of teens found that those currently participating in sport reported higher well-being and more favorable school outcomes, including less perceived school stress and a greater sense of belonging at school, compared to those who had dropped out of sport (11). International research similarly shows that youth who play sports tend to have higher self-esteem and a lower likelihood of engaging in risky behaviors. For many children, the team environment makes exercise fun and socially rewarding, thereby sustaining their involvement in physical activity. Sports can also impart lifelong healthy habits and skills like teamwork and discipline. Because of these benefits, we believe that expanding access to organized sport could be a key strategy in combating the decline in youth fitness.

However, we must also acknowledge that organized sport is not the only solution and that youth sports are facing a decline in participation and inclusivity, especially during adolescence. Across many countries, there is a well-documented trend of youngsters quitting organized sports as they enter their mid-teen years. In the United States, for example, it has been estimated that around 70% of kids drop out of youth sports by age 13 (15). Norway mirrors this pattern: a national report by Norwegian Social Research (NOVA) noted that over half of adolescents who were active in sports at the start of their teens had quit by age 17 (16). The reasons are multi-faceted and shaped by interacting psychological, social, and structural factors. A review uncovered consistent declines in participation during adolescence across European countries, particularly between ages 14 and 18 (17). Findings revealed that drop-out is often associated with negative sport experiences, changing life priorities, and organizational demands. Hence, understanding sport drop-out requires a contextualized perspective that situates individual decisions within wider social and structural conditions. On one hand, academic pressures and new interests emerge in the teenage years, often squeezing out time for sports. On the other hand, youth sports structures can inadvertently drive adolescents away through early specialization, high costs, and competitive pressure. Many sports programs become increasingly performance-oriented in adolescence, emphasizing elite pathways and early talent selection. Teenagers who simply want a more casual, play-for-fun experience may feel there is no place for them and thus drop out. This is exacerbated by the practice of early selection, for instance, streaming elite squads at ages as young as 9–10, which can demotivate those who are labeled less talented or less developed. Burnout and injuries from year-round specialization in a single sport may also contribute to the drop-out problem (18). Additionally, social disparities limit who stays in sports. Gender gaps are significant: girls tend to leave sports sooner and in greater numbers than boys, often citing lower enjoyment or confidence. By age 15, many countries see a sharp drop in girls' sports participation, reflecting a mix of cultural norms and inadequate support or role models for female athletes (7).

Economic barriers further stratify sports participation. As sports programs demand more expensive equipment, travel, or training, youth from lower-income families often cannot keep up. A recent analysis in Norway confirmed that adolescents still active in sports had significantly higher family socioeconomic status (SES) on average than those who had quit, and the drop-out rate was as high as 80% among youth from the lowest SES group (11). All the abovementioned factors mean that while organized sport is a vital arena for physical activity, it currently fails to engage all youth equally or retain them through adolescence. The young people most in need of regular activity, those from disadvantaged backgrounds or those less naturally inclined to sports, are often the ones falling through the cracks of the sports system.

In this context, self-organized (unorganized) forms of participation represent an important dimension of sport engagement in adolescence. These activities are typically characterized by greater autonomy and flexibility, have been shown to support developmental outcomes including intrinsic motivation and social competence. From our point of view, self-organized and organized participation should be viewed as complementary rather than mutually components, with evidence suggesting that combined engagement is associated with particularly positive developmental profiles (19). In addition, the growing imbalance between organized sport participation and unstructured free play in youth populations is a major concern. This pattern is influenced by environmental and policy-level factors, including urban design and traffic-calming initiatives, which can either facilitate or restrict opportunities for unorganized physical activity.

The Norwegian sports model (aiming to include “as many as possible for as long as possible”) offers an illuminating case study in both the potential and the challenges of using organized sport to promote youth fitness. Norway is known for its high sports participation rates: upwards of 90% of Norwegian children take part in a sports club at some point during their childhood (20). This is facilitated by a widespread network of local sports clubs, significant public funding, and cultural emphasis on volunteer-led youth sports. Notably, Norway's approach has long prioritized children's enjoyment and broad inclusion. National guidelines discourage formal competition and ranking for kids, aiming to prevent early exclusion and keep the door open for late bloomers (20). The result has been a robust pipeline of both recreational athletes and world-class champions. It is no coincidence that Norway, despite its small population, often excels in international sports competitions, a fact many observers attribute to its large base of active youth and the deliberate delay of specialization. In principle, Norway demonstrates that it is possible to combine mass participation with elite success by maintaining a broad grassroots foundation. This approach has scientific support, as a recent analytical review shows that nine out of ten world-class athletes across sport disciplines did not peak early, indicating the need for a patient and sustainable developmental approach (21). In addition, research shows that world-class performance is most often achieved gradually through broader multidisciplinary practice during adolescence (21). Despite this evidence, elite programs for young children continue to emerge and are often structured to prioritize short-term performance.

Currently, the Norwegian system also faces pressures. Debates highlight early talent selection and strong parental involvement challenging the children-first ethos. Despite policies, some environments prioritize early selection and winning, risking reduced inclusion. Norway also sees a sharp mid-teen drop-out, with over half leaving organised sport by 17, due to academic demands, other interests, costs, and exclusion. Performance pressure persists, as elite ambitions can conflict with sport-for-all ideals. While generally affordable, some sports become costly at higher levels. Overall, despite high participation, Norway struggles to retain adolescents, suggesting that sports clubs alone are insufficient and that broader strategies are needed.

## 4 Bridging the gap through the education system

One promising avenue is to integrate sport and physical activity more deeply into the education system, essentially, bringing the “sport for all” philosophy into schools. Schools are the one institution that nearly all children attend, regardless of background or interest, so they present a unique opportunity to deliver physical activity regularly to every child. Schools are suggested to provide an ideal setting to promote both health and wellbeing (22). Moreover, schools represent complex socio-ecological environments shaping young people's health behaviors (23). From a socio-ecological perspective, health behaviors are shaped through interactions across multiple levels, including intrapersonal, interpersonal, institutional, and policy domains, highlighting the importance of considering both in-school and out-of-school influences. In a recent discourse (not peer-reviewed), we have suggested that “*the next generation’s Norwegian sports model starts in the classroom,”* meaning that daily physical activity in schools should form the backbone of youth fitness efforts, complementing after-school sports clubs (24). This concept is gaining traction as a response to the decline in free play and the rise in youth sports drop-out. The idea is that every school day should include a substantial dose of physical activity, whether through daily physical education (PE) classes, active recess and breaks, or even integration of movement into academic lessons. If implemented, such a policy would guarantee that all children, even those who are not in any sports club, get regular exercise and develop basic physical literacy. Norway has already piloted aspects of this approach: for instance, positive effects of a 2-year school-based PA intervention remained for cardiovascular health risk factors 5 years after cessation of the intervention (25) and an innovative model at a Norwegian sports-oriented middle schools have incorporated training as part of the daily schedule for nearly 20 years, reportedly with excellent results in student fitness and engagement.

The challenge now is to extend similar principles to typical public schools and younger age groups. This requires a paradigm shift, and we propose that physical activity should not be treated as an extracurricular option but as a core component of education. According to the authors own perspective, physical activity should be as fundamental as math or reading in the school system. It also requires creative collaboration across sectors: educators, public health officials, and sports organizations working together. In practice, we propose that schools should partner with local sports clubs to offer after-school programs on the school premises, use club coaches to assist PE teachers or run sports clinics, and make school facilities available for community sports in off-hours. Offering sports or other organized activities (e.g., group-based functional training) that are not centered on competition may be essential, as competitive pressure is frequently cited as a reason why adolescents disengage from organized sport. Providing broader, low-threshold opportunities that emphasize social belonging and participation may therefore play a key role in promoting higher levels of physical activity. Such school–sport collaborations can lower practical barriers (like transportation or cost) that often prevent kids from participating. Importantly, a school-based strategy can be more inclusive: activities can be tailored for all skill levels and interests, from traditional team sports to dance or outdoor pursuits, ensuring each child finds a form of movement they enjoy. When done thoughtfully, making physical activity part of the school culture may also enhance academic outcomes, there is evidence that regular activity improves concentration and cognitive function in students (26, 27). In essence, a comprehensive school-centric approach could address the gaps left by the club system, reaching children who would otherwise be inactive and helping to sustain activity for those who leave competitive sports.

Implementing such changes on a broad scale will require overcoming certain barriers. One is the perception of trade-offs in the school day: educators and parents may worry that more time spent moving will mean less time for academics. Policymakers will need to emphasize that physical health and academic performance can complement each other, as active, healthy students often learn better. Another barrier is resource constraints: schools, especially in under-resourced areas, may need additional support (funding for equipment, training for teachers) to incorporate daily activity. Moreover, fostering collaboration between the education and sports sectors means bridging organizational cultures; it takes strong leadership and clear incentives to get schools and clubs to work together effectively. Economic factors cannot be ignored: while moving activity into schools can reduce direct costs for families, it still requires public investment. Daily implementation of physical activity in schools may also include potential challenges related to logistics or alignment of the curricular, in addition to resource-related barriers, as well as the need for context-specific implementation strategies. Despite these challenges, the opportunities are immense and successful implementation will depend on local educational contexts and policy priorities. If every child gains the habit of daily exercise through school, it could instill a foundation of fitness and well-being that carries into adulthood, regardless of whether they participate in formal sports. It might also feed back into sports recruitment, as active kids at school become more inclined to join local teams. In the long run, a physically active generation would ease future healthcare burdens and yield a more productive, healthier society. Despite schools providing an essential setting for physical activity promotion, increasing opportunities alone may be insufficient to increase participation. Therefore, broader engagement is needed from an ecological perspective, including family resources, transportation, competing leisure activities, and other environmental and social factors that shape children's access to and participation in physical activity.

In confronting the youth fitness crisis, we face a paradox: the scientific knowledge on how to combat it is largely in hand, yet society has been slow to act on this knowledge at the needed scale. The health benefits of exercise are unequivocal, and we have examples, like effective school-based activity programs, that demonstrate feasible ways to get young people moving. Why, then, are inactivity and obesity rates still climbing? We believe that part of the answer lies in modern lifestyles and systems that have not prioritized physical activity. Despite that other factors such as dietary intake also warrant consideration (28), physical activity remains highly important for metabolic health and weight regulation. Poor dietary habits and increasing rates of childhood obesity may create additional barriers to physical activity participation, highlighting the need for integrated policies that support healthy lifestyles among children and adolescents.

Additionally, the issue cuts across sectors (health, education, sports, urban planning), making it challenging to coordinate. However, recognizing this inertia is the first step to overcoming it. Governments might consider mandating daily physical education in schools, supported by national curricula that emphasize inclusive, enjoyable activities for all. Policies to reduce economic barriers in youth sports are also needed, such as subsidies to lower participation costs and investments in community sports facilities to ensure access in underserved areas. Equally important is training and incentivizing schools and clubs to collaborate, blending their reach and expertise. On an international level, countries should share best practices and learn from each other's successes and failures, adapting strategies to their own cultural contexts.

## 5 Limitations

Some of the proposed solutions are context-dependent, which means that caution should be exercised when considering the transferability of the Norwegian model to other countries with different cultural, organizational, and policy environments. Furthermore, as a perspective article, this work does not rely on systematic empirical methods, which may limit the generalizability and evidential strength of the arguments presented. Our suggestions and narrative are based on selected literature and the Norwegian contextual nature, and thus, may not always be generalized due to selection bias. For example, one opinion paper [not peer-reviewed; (24)] is used solely as an illustrative example of the broader public discussion.

## 6 Future outlook

Slowing and reversing the decline in youth fitness is a global imperative. One path forward may involve revitalizing organized sports to go beyond the club model by making physical activity a daily norm in schools. We argue that youth inactivity will require a broader, more inclusive approach that goes beyond the sports club model, and propose integrating substantial daily physical activity into school curricula as a universal foundation for fitness and health. By reimagining schools as key platforms for physical activity—in partnership with community sports programs—and by implementing bold, multi-sector policies to reduce barriers to active lifestyles, society can begin to bridge the gap between what we know and what we practice. Ultimately, we must bridge the gap between knowing what needs to be done and actually doing it. Ensuring that children and adolescents have opportunities and encouragement to be active every day is an achievable goal with the knowledge and tools already at our disposal.

## Data Availability

The original contributions presented in the study are included in the article/Supplementary Material, further inquiries can be directed to the corresponding author.
